# An authoritarianism-compatible text changes British attitudes towards EU immigration

**DOI:** 10.1038/s41598-025-11491-z

**Published:** 2025-09-12

**Authors:** Tessa Buchanan, David J. Young, James Ackland, Alan Renwick, Lee de-Wit

**Affiliations:** 1 Political Psychology Lab, University of Cambridge, Cambridge, UK; 2 Democratic Politics, Constitution Unit, University College London, London, UK

**Keywords:** Psychology, Human behaviour

## Abstract

Immigration was a major point of contention for voters in 2024, a record year for elections. Here we test whether British attitudes towards immigration become more positive when participants are exposed to a framed text. In the study (n = 3067), which uses a sample sourced from YouGov that is representative for age, education, gender and politics, participants are exposed to a short text written to be compatible with moderate levels of what political psychologists call ‘authoritarianism’ that also incorporates factual arguments and an emotional appeal. We find that people exposed to this text as opposed to a control text or a low authoritarianism text feel they share more values with a fictitious EU immigrant, and are more positive towards EU immigration. There are also differences in overall immigration attitudes after reading the authoritarianism-compatible text relative to the control, but these are smaller, and do not differ significantly from the low authoritarianism text. These findings demonstrate that persuasion is possible on contentious issues like immigration, and are consistent with the idea that authoritarianism-compatible arguments might be particularly effective for culturally similar forms of immigration (such as EU immigration to the UK).

## Introduction

Billions of people went to the polls in 2024, a record year for elections. In richer regions, such as the USA and Europe, immigration proved to be a key area of contention^1–3^, much as it has been in electoral contests throughout the last decade. Whilst there are advantages and disadvantages to immigration, there is a perception that people are unlikely to be convinced or persuaded by arguments about the positives of immigration. As an example, during the 2016 EU referendum debate, then UK Prime Minister David Cameron felt it was a subject on which he had “no clear answer”^4^.

In the psychology literature, a 2021 meta-analysis^5^ found that there were not enough actionable, evidence-based recommendations for reducing prejudice, including that directed against immigrants. The authors of that study recommended that scholars investigate older, more complex and powerful psychological forces based around social norms, group dynamics, authority and hierarchy.

Looking at the correlates of immigration attitudes in the UK, a strong association exists with a psychological construct known as authoritarianism^6^. Those high in authoritarianism tend to be opposed to immigration and those low in authoritarianism tend to be in favour of it^7^.

In political science, the term ‘authoritarian’ is commonly used to describe anti-democratic regimes or leadership styles^8^. However, in the political psychology literature, ‘authoritarianism’ is a term used to describe an enduring psychological characteristic.

Psychologists first began to study authoritarianism in the context of World War II^9^. The earliest researchers conceived of it as an umbrella-type construct^10^. They associated it with prejudice against outgroups; a tendency to think in black-and-white terms; an enhanced sensitivity to cleanliness; a strong preference for the in-group and its rules and norms; and a desire for a strong leader and an orderly society^11^. In 1981, three sub-dimensions were identified: authoritarian aggression, submission and conventionalism^12^.

More recent research^13^ suggests that some people have a predisposition towards authoritarianism and will adopt authoritarian behaviours and attitudes when exposed to ‘normative threat,’ or the feeling that their group might be in danger. This sense of threat might be sparked by doubts in the competence of the authorities, disrespect for leaders, a lack of conformity with group norms or polarisation^14^. Conversely, such people might respond to ‘normative reassurance’ if persuaded that their preferred sense of oneness and sameness is being restored^15^.

Authoritarianism has known associations with other psychological characteristics. High levels of authoritarianism are associated with the Big Five^16^ personality trait of Conscientiousness (as opposed to Openness^17^); the “binding” Moral Foundations^18^ (Authority, Loyalty and Purity^19^); the Schwartz^20^ values of Conservation (Conformity, Tradition and Security) as opposed to the Openness values of Stimulation and Self-Direction^21^; cognitive inflexibility^22^, disgust^23,24^ and social conformity^25^.

Taken together, this broad literature provides indications as to the type of language or themes that might appeal to those high and low in authoritarianism. Given that framing short texts to match the psychological characteristics of the audience has been shown to be an effective persuasion technique^26^, the literature suggests what elements such texts might contain.

In this study, the research question was whether attitudes towards immigration could be made more positive. We decided to target an audience who were likely to be opposed to immigration – those scoring in the top 50% for authoritarianism. However, there were gradations to consider. Those scoring in the very highest centiles for authoritarianism would include people who were likely to support aggressive, punitive measures and harsh treatment for non-conforming out-groups. Their views might alienate those with more centrist opinions, entrenching rather than bridging moral divides^27^. For this reason, we aimed to draft a text that would be compatible with moderately authoritarian views such as respect for order, tradition, social norms, hard work and hygiene.

For comparison, we also drafted a text aimed at those low in authoritarianism, which celebrated diversity, creativity and the arts.

## Methods

Two pilot studies (n = 2004, n = 1006) were conducted using Prolific Academic to refine the texts and methodology.

For the main experiment, the polling company YouGov recruited 3067 participants, and data were collected from 7 to 12 March 2023. Three texts of about 400 words were drafted. The control text was composed by ChatGPT in order to avoid experimenter bias. It discussed bread quality, which we regarded as a neutral topic. The first treatment text was drafted to be compatible with moderate levels of authoritarianism. The second treatment text, included for comparison, was drafted to appeal to those with low levels of authoritarianism.

There were common elements between the two treatment texts. Both discussed a fictitious immigrant from Poland, who was named as ‘Sonia’. She was described as someone who had come to the UK five years ago, who would like to remain in the country and had applied for British citizenship.

Factual information was kept the same between the two texts. We included arguments based on reputable sources. The text cited the Office for Budget Responsibility^28^ on the long-term contribution made by immigrants to the UK’s finances; the Office for National Statistics^29^ on their contribution to the National Health Service (NHS); and “figures quoted by the government”^30^ which show that European Union immigrants make a particularly positive contribution in terms of the tax they pay.

Both texts ended with the same sentence—an emotional appeal touching on the immigrant’s concern for the future and whether she was still welcome in the UK.

However, there were notable differences to reflect the two framings. The authoritarianism-compatible text included a social norm saying that most British people supported skilled immigrants coming to the UK. It expressed concern about the chaos that ensues when essential jobs go unfilled, and it described ‘Sonia’ as a nurse, who valued safety, fairness, duty and hard work. For her, the late Queen Elizabeth II’s Platinum Jubilee celebrations were a recognition of the contribution made by those staffing hospitals, schools and essential services.

The low authoritarianism text stressed the contribution made by immigrants towards diversity, culture and education. ‘Sonia’ was described as an entrepreneurial fashion designer, who felt that race and religion were becoming ever less important, and who enjoyed seeing Bollywood and drag queens celebrated during the Jubilee events.

We pre-registered the following hypotheses on the Open Science Framework, reflecting our primary focus, which was whether attitudes towards immigration could be changed at all.

### Hypothesis 1

Those exposed to the authoritarianism-compatible text will feel closer to the immigrant than those exposed to a control.

### Hypothesis 2

Those exposed to the authoritarianism-compatible text will be more positive about EU immigration than those exposed to a control.

### Hypothesis 3

Those exposed to the authoritarianism-compatible text will be more positive about immigration than those exposed to a control.

Six additional hypotheses (Hypotheses 4–9) were pre-registered that, for each of these three categories, those high in authoritarianism would respond more positively when exposed to the authoritarianism-compatible text than when exposed to the low authoritarianism text, and those low in authoritarianism would respond more positively when exposed to the low authoritarianism text as compared to the authoritarianism-compatible text.

Respondents to the YouGov survey answered two political questions, and two short surveys testing for Authoritarianism and Social Dominance Orientation. They were then randomly assigned to one of three groups. Those in the control group read the neutral text on bread quality while those in the treatment groups read either the low authoritarianism text or the authoritarianism-compatible text.

Participants were then asked to respond to four questions. The first tested for shared values; the second asked whether the individual felt EU immigration was a good or a bad thing for the UK; the third was a question as to whether they felt there were too many or too few immigrants in the UK (the “immigration stock” question), and the fourth was a question as to whether more or fewer immigrants should be let into the country (the “immigration flow”) question.

## Results

### Comparing the authoritarianism-compatible text and the control

On the values question, there was a significant difference between those exposed to the control (“Cont”) text (M = 4.07, SD = 1.56) and those exposed to the authoritarianism-compatible (“AC”) text (M = 5.13, SD = 1.56): (t = – 15, df = 2039, p < 0.001, conf. int. [– 1.19, – 0.92], 

d = – 0.68), who reported sharing more values with the fictitious immigrant.

On the EU immigration question, there was a significant difference between the responses of those exposed to the two texts (“Cont” M = 3.41, SD = 1.83; “AC” M = 2.61, SD = 1.61; t = 10.24; df = 1901, p < 0.001, conf. int. [0.65, 0.95], d = 0.46), with those exposed to the authoritarianism-compatible text feeling more positive about EU immigration.

Additionally, those exposed to the authoritarianism-compatible text were significantly more positive on both immigration stock (“Cont” M = 3.79, SD = 2.05; “AC” M = 4.13, SD = 1.98; t = – 3.7, df = 2030, p < 0.001, conf. int. [– 0.51, – 0.16], d = – 0.16) and immigration flow (“Cont” M = 4.52, SD = 1.78; “AC” M = 4.21, SD = 1.75; t = 4.0, df = 2035, p < 0.001, conf. int. [0.16, 0.46], d = 0.18) when compared to the control group, although the effect size for these variables was relatively small. It should be noted that our pre-registration combined the stock and flow measures, but given the consistency of the results, and the high correlation between these two outcomes, we decided to report the results for both outcomes (for transparency, the supplementary materials to this article contain the results with the combined measure).

### Comparing the low authoritarianism text and the control

The low authoritarianism text was then compared to the control to see if arguments based around diversity would similarly shift attitudes.

Those exposed to this text were significantly more positive than the control group on the values question (“Cont” M = 4.07, SD = 1.56; “LA” M = 4.47, SD = 1.71; t = – 5.47, df = 2010, p < 0.001, conf int [– 0.54, – 0.26], d = – 0.24); on the EU immigration question (“Cont” M = 3.41, SD = 1.83; “LA” M = 2.93, SD = 1.72; t = 6.03, df = 1930, p < 0.001, conf. int [0.33, 0.64], d = 0.27); on the immigration stock question (“Cont” M = 3.79, SD = 2.05; “LA” M = 4.05, SD = 2.05; t = – 2.84, df = 2018; p = 0.004, conf int [– 0.44, – 0.08], d = – 0.13) and on the immigration flow question (“Cont” M = 4.52, SD = 1.78; “LA” M = 4.30, SD = 1.82; t = 2.80, df = 2019, p = 0.005, conf int[0.07, 0.38], d = 0.12).

**Fig. 1:**
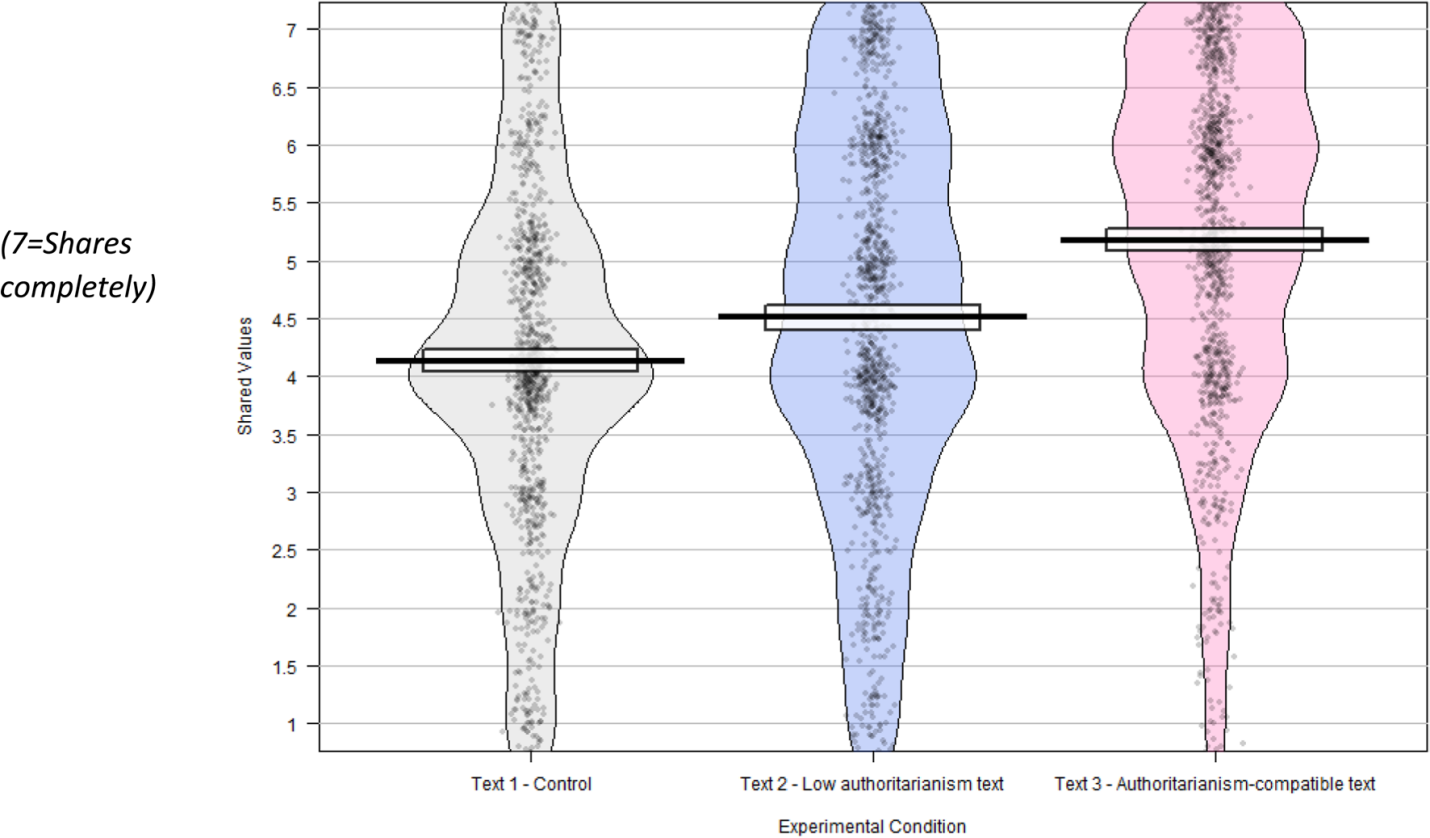
To what extent does the migrant mentioned in the text share or not share your values? N = 3067, weighted to be representative for age, education, gender and politics.

**Fig. 2:**
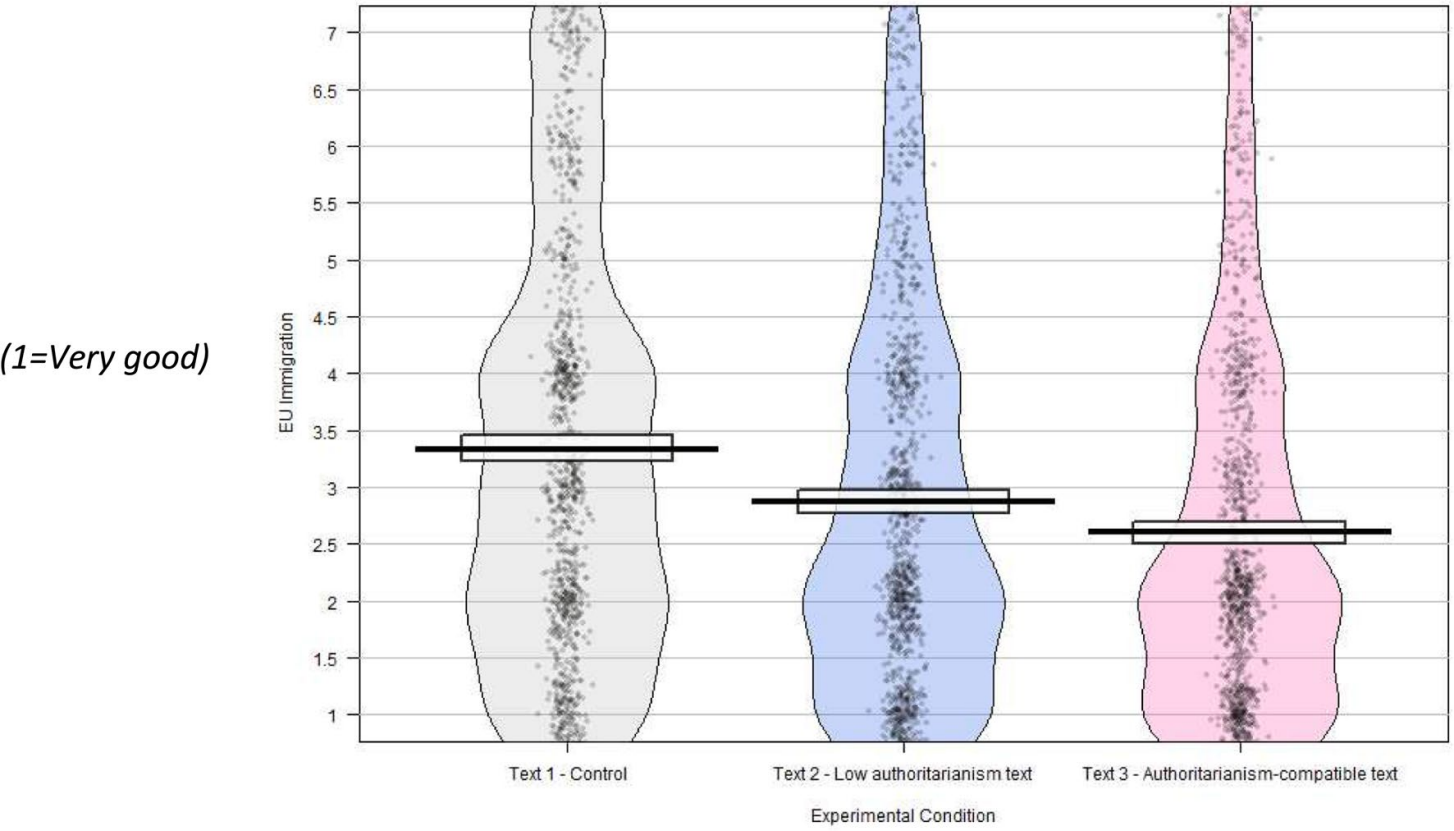
EU immigration—good or bad for UK? N = 2987, weighted to be representative for age, education, gender and politics with “Don’t Know” responses removed.

**Fig. 3:**
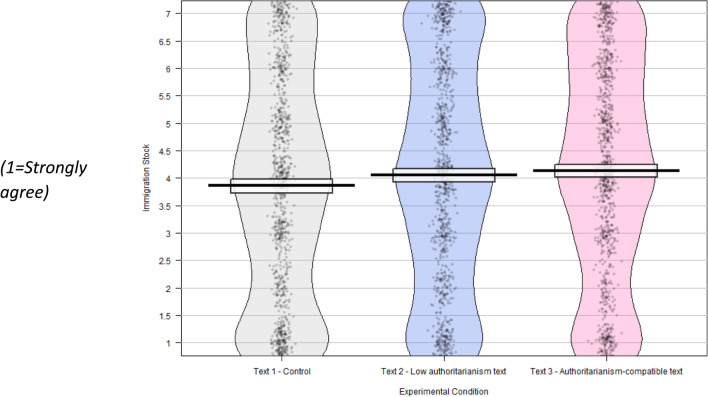
Immigration stock—do you agree or disagree there are too many migrants in UK? N = 3067, weighted to be representative for age, education, gender and politics.

**Fig. 4:**
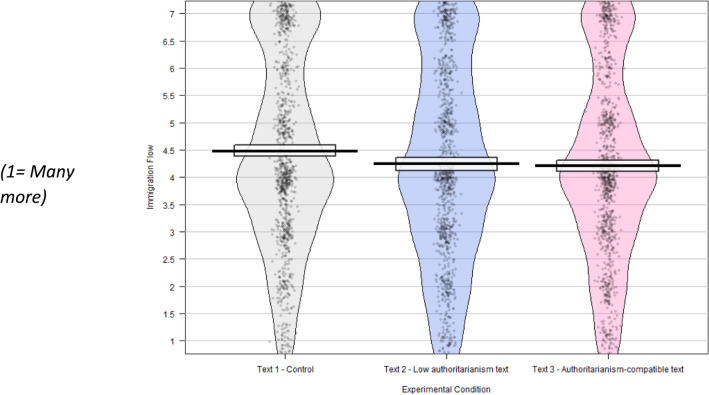
Immigration flow—allow many more or many fewer migrants to UK? N = 3067, weighted to be representative for age, education, gender and politics.

While the attitudes of those exposed to the low authoritarianism text were more positive than for those exposed to the control text, the effect sizes were smaller than for the authoritarianism-compatible text, as illustrated in Figs. 1, 2, 3, 4.

### Comparing the authoritarianism-compatible and the low authoritarianism texts

Finally, two sets of post-hoc tests were carried out to compare the results for the authoritarianism-compatible text directly with those for the low authoritarianism text.

For the first set of tests, t-tests were used. Those exposed to the authoritarianism-compatible text reported that they shared significantly more values with the fictitious immigrant than those exposed to the low authoritarianism text (“AC” M = 5.13, SD = 1.56; “LA” M = 4.47, SD = 1.71; t = – 9.15, df = 2042, p < 0.001, conf. int. [– 0.80, – 0.52], d = – 0.40). They also felt more positive about EU immigration (“AC” M = 2.61, SD = 1.61; “LA” M = 2.93, SD = 1.72, t = 4.22; df = 2010, p < 0.001, conf. int. [0.17, 0.46], d = 0.19). However, there was no significant difference on the immigration stock variable (“AC” M = 4.13, SD = 1.98; “LA” M = 4.05, SD = 2.05; t = – 0.82, df = 2060, p = 0.41, conf. int. [– 0.25, 0.10], d = – 0.04) nor on the immigration flow variable (“AC” M = 4.21, SD = 1.75; “LA” M = 4.30, SD = 1.82; t = 1.12, df = 2057, p = 0.26, conf. int. [– 0.07, 0.24], d = 0.05).

The second test involved 2 × 2 anovas, which were used to investigate whether people who scored high or low for authoritarianism responded differently to the two texts, as per the additional Hypotheses 4–9.

We found that for the first item – the extent to which participants felt that they shared values with the fictitious immigrant – there was a significant main effect of the participant’s level of authoritarianism (F(1,2064) = 71.4, p < 0.001), and a significant main effect of the text that they were exposed to (F(1,2064) = 90.6, p < 0.001). Additionally, there was a significant interaction between the participant’s level of authoritarianism and the text they were exposed to (F(1,2064) = 9.7, p = 0.002). Those higher in authoritarianism responded more positively after being exposed to the authoritarianism-compatible text rather than to the low authoritarianism text.

On the second variable – attitudes towards EU immigration – the analysis showed a significant main effect of the participant’s level of authoritarianism (F(1,2027) = 244.6, p < 0.001) and a significant main effect of the text that the participant was exposed to (F(1,2027) = 15.9, p < 0.001). However, the interaction between the participant’s levels of authoritarianism and the text that they were exposed to was not statistically significant (F(1,2027) = 1.7, p = 0.19). This suggests that the levels of authoritarianism and the choice of text independently influenced attitudes towards EU immigration.

For the immigration stock variable, there was a significant main effect of the participant’s level of authoritarianism (F(1,2064) = 460.9, p < 0.001). However, there was no significant main effect of the choice of text (F(1,2064) = 1.2, p = 0.27), nor any significant interaction between the level of authoritarianism and the choice of text (F(1,2064) = 0.05, p = 0.83). Similarly, for the immigration flow variable, there was a significant main effect of the level of authoritarianism (F(1,2064) = 460.9, p < 0.001), but the main effect of the text that the participant was exposed to (F(1,2064) = 1.24, p = 0.27) and the interaction effect (F(1,2064) = 0.05, p = 0.83) were insignificant.

Graphs have been included in the supplementary material to illustrate these findings. When responses are mapped against the participants’ scores for authoritarianism, we found non-linear results.

## Discussion

In 2025, immigration remains one of the most controversial topics in politics. The new administration of Donald Trump has made securing the US borders a key priority^31^, and the government led by Sir Keir Starmer is likewise keen to reduce the numbers of illegal immigrants heading to the UK^32^. Immigration was a major factor in the German federal elections in February^33^.

It was outside of the scope of this work to evaluate what level of immigration is appropriate for any given country. Across any population, one can assume that there would be a range of views from the most liberal to the most restrictive. Careful consideration would be needed to determine the context and individual circumstances in which it is ethical to use persuasive messages to change attitudes on immigration, including any potential secondary effects of using language that might be regarded as moralised or of reinforcing ideas that are compatible with moderate levels of authoritarianism, such as respect for order, tradition, social norms, hard work and hygiene. Our primary contribution is to demonstrate that attitudes towards immigration can change, and can indeed become more positive in some contexts. With a large sample sourced from YouGov, we found that these effects could be observed after a relatively modest intervention in which British participants were asked to read about 400 words.

We drafted three texts for our experiment: a control text; a text framed to reflect the values of those with low levels of authoritarianism; and a text framed to be compatible with moderate levels of authoritarianism. Both treatment texts included factual material and an emotional appeal.

When participants were exposed to the authoritarianism-compatible text as opposed to a control, they reported sharing more values with a fictitious Polish immigrant and there was a pronounced change in their attitudes towards EU immigration. In the control condition, a total of 53% said that EU immigration was good for the UK (i.e. “Slightly good”, “Moderately good” or a “Very good thing”) while for those who read the authoritarianism-compatible text, the equivalent percentage was 73%, some 20 percentage points higher. Participants were also slightly more positive about immigration overall, although these results were less clear than for the other measures.

A secondary contribution made by this study is that it demonstrates that some forms of argument are more effective than others. Exposure to both treatment texts generated a positive effect as compared to the control, but while the low authoritarianism text did better than anticipated, the strongest effects were observed when participants were exposed to the authoritarianism-compatible text. Both texts focused on an individual since research shows that it is easier to empathise with a single person than with many^34^. In the authoritarianism-compatible text, the fictitious Polish immigrant was framed in terms that were designed to reassure people. She was engaged in a caring occupation and upheld traditional values. By contrast, in the low authoritarianism text, she was a creative who enjoyed the freedom and diversity offered by the UK.

When the two treatment texts were compared to each other, the authoritarianism-compatible text out-performed the low authoritarianism text on two measures – the values variable and attitudes towards EU immigration. We also found that there was an interaction on the values measure, suggesting that those high in authoritarianism preferred the authoritarianism-compatible text. These results can be seen in more detail in the supplementary material and data provided.

The factual material and emotional appeals that were common to both treatment texts may also have contributed to the change in attitudes. Looking at the immigration stock and flow measures, it was not possible to distinguish between the results for the two treatment texts on these measures but both produced slightly more positive results than the control text.

As a theoretical contribution, the authoritarianism-compatible text brought together elements that are sometimes tested in isolation, such as social norms messaging or messages framed in terms of particular personality traits (in this case, Conscientiousness). In this study, we constructed a persuasive text that is consistent with theorising that sees authoritarianism as an umbrella-type construct under which other psychological characteristics are grouped together in predictable ways.

Building on these findings, further investigations would be needed to see if these results could be replicated and consolidated; to test the effectiveness of factual arguments; and to explore whether targeting communications based on levels of authoritarianism is an avenue that merits further study. Researchers may also wish to explore in greater depth whether an approach to messaging which combines different elements is more effective than using elements individually to frame messages. Our finding is consistent with the idea that the authoritarianism-compatible framing might be more effective for more culturally similar migrant groups (like EU migrants), although this would need to be tested more explicitly in future research. The factual material in this experiment made direct reference to the impact of EU immigration on the UK. If the experiment were repeated to consider migrants from more culturally distant countries, then different factual material would be required and the participants’ views might be shaped by the extent to which they felt such immigrants would successfully integrate into the UK.

Summarising, we have shown that arguments combining shared values, evidence from trusted sources of information and an emotional appeal can change British attitudes towards immigration, and EU immigration in particular. This builds towards a body of evidence that people can be persuaded by arguments incorporating reason when these are carefully constructed.

In a world where immigration remains politically controversial and where climate-driven migration is rising, we think this work offers important insights for policy-makers and politicians in understanding what drives attitudes towards immigration and what types of argument might be effective in changing those attitudes.

## Procedure

### Participants

YouGov recruited 3067 participants (1706 women) with an average age of 50. YouGov supplied weights to make the survey representative for age, education, gender and politics. Each participant was paid 50 YouGov points for taking part in the 5-min survey.

### Procedure

After being asked for consent, participants were asked who they would vote for if a general election were held tomorrow, and if they thought the process of the UK leaving the EU (Brexit) was going well or badly. Exact responses are recorded in the documentation on the Open Science Framework. They reflected contemporaneous polls.

Participants responded to the 6-item Very Short Authoritarianism (VSA) scale^35^ and the 4-item SDO scale^36^. They were then randomly split into three groups and exposed to either the control or one of the treatment texts.

### Variables

**Fig. 5:**
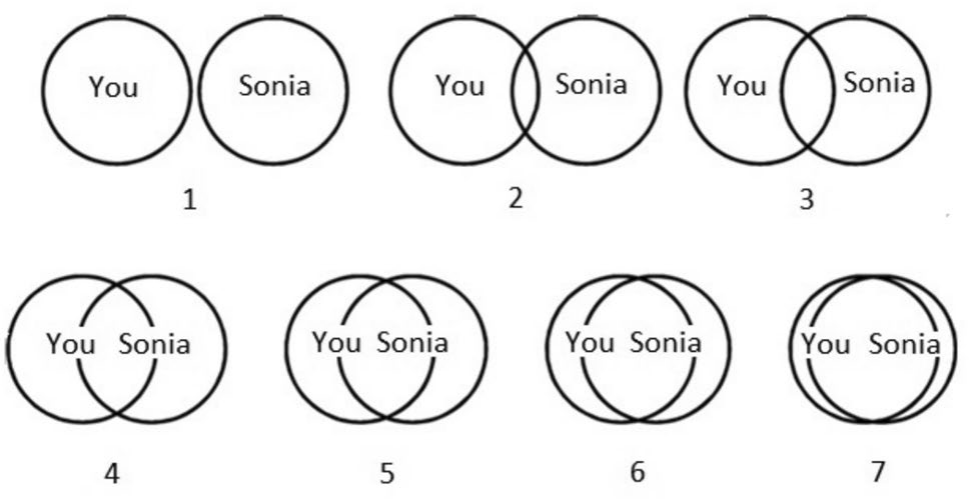
The shared values variable.

The first dependent variable asked participants to choose which of seven increasingly overlapping circles best described the extent to which the fictitious immigrant shared their values (Fig. 5).

These circles, numbered from 1 to 7, were based on the ‘Inclusion of Other in Self’ scale^37^. Since those exposed to the control condition would have very little detail about the immigrant, respondents were encouraged to go with their gut instinct. The exact wording was: “Please select the pair of circles that best describes the extent to which Sonia shares or doesn’t share your values. Please answer this question quickly. If you’re not sure, or if there is not enough information, it’s best to go with your gut instinct.” The responses were recorded on a 1–7 Likert scale. To avoid confusion, the score of 1 was additionally marked as “Doesn’t share at all” and a score of 7 was marked as “Shares completely”.

EU immigration attitudes were tested with the following question: “Do you think immigration from EU countries is a good or bad thing for the UK?” A seven-point scale was used to avoid a potential ceiling effect. The response options were 1 = “Very good thing”, 2 = “Moderately good”, 3 = “Slightly good”, 4 = “Neither good nor bad”, 5 = “Slightly bad”, 6 = “Moderately bad”, 7 = “Very bad”, 8 = “Don’t know”. Those who responded “Don’t know” (3% of the total sample) were excluded from the analysis.

Attitudes towards the stock of immigrants were measured with the following question with responses recorded on a 7-point Likert scale: “To what extent do you agree or disagree with the following statement: “There are too many immigrants in the UK right now”, with scores ranging from 1 = “Strongly agree” to 7 = “Strongly disagree”.

The fourth dependent variable measured attitudes towards the flow of immigrants. It was worded as follows: “Some people think that the UK should allow many more immigrants to come to the UK to live, and others think that the UK should allow fewer immigrants. Where would you place yourself on this scale?”. Responses were measured on a 7-point Likert scale ranging from 1 = “Many more” to 7 = “Many fewer”.

At the end of the survey, participants were thanked and debriefed.

## Supplementary Information

Supplementary Information.

## Data Availability

The pre-registration, data and code supporting the findings in this research can be found on the Open Science Framework on this link: https://osf.io/hbu5x/ .
