# Supplementary material for: An authoritarianism-compatible text changes British attitudes towards EU immigration
Source: Sci Rep. 2025 Sep 12;15:31724. doi: 10.1038/s41598-025-11491-z (PMC12432221; doi:10.1038/s41598-025-11491-z)

**An authoritarianism-compatible text changes British attitudes towards EU immigration**

**Buchanan et al. (2025) - Additional notes**

**Pre-registration**

The pre-registration can be found on this OSF link <https://osf.io/hbu5x/>.

The pre-registration said that immigration attitudes would be tested by using a score that combined the immigration stock and flow variables. In the published paper, these figures are given separately, but for transparency and completeness, the scores for the composite measure were as follows:

**Control v. authoritarianism-compatible texts**

A composite immigration score was created from averaging the responses to the immigration stock and flow questions. There was a significant difference in the responses between those exposed to the authoritarianism-compatible and the control texts (“Cont” M=3.64, SD=1.81; “AC” M=3.96, SD=1.77; t=-4.06, df=2033, p<.001, conf. int [-.47, -.17], d=-.18).

**Control v low authoritarianism texts**

There were significant differences on the composite immigration score between the responses of those exposed to the control text and those exposed to the low authoritarianism text (“Cont” M=3.64, SD=1.81; “LA” M=3.88, SD=1.84; t=-2.98, df=2019, p=.003, conf int [-.40, -.08], d=-.13).

**Data and code**

The data and code for this article can be found on this OSF link <https://osf.io/hbu5x/> .

For the first pilot study (Study 1), please use the data files: Remain.csv, Leave.csv and Control.csv. The code is labelled as “Code for Study 1 – final.R”. The authoritarianism-compatible text is labelled “Gen” and the control is labelled as “Control”.

For the second pilot study (Study 2), please use the data files: LongtextLeave.csv, LongtextRemain.csv, Control.csv, ControlEUFunkeLeave.csv, ControlEUFunkeRemain.csv. The code is labelled as “Code for Study 2 – final.R”.

For the main experiment, please use the data YGnumeric.csv. The code is labelled as “Code for Study 3 – final.R”.

**Experimental texts:**

Text 1: ChatGPT text used for the control

Bread is a staple food that has been consumed by people for thousands of years. It is a simple food made from a few basic ingredients, but the quality of the bread can vary greatly depending on how it is made, the ingredients used, and the conditions in which it is baked.

Bread quality is an important consideration for both bakers and consumers. For bakers, the quality of their bread can determine the success of their business, while for consumers, such as Sonia from Poland, the quality of the bread can affect its taste, texture, and nutritional value.

One of the key factors that contribute to the quality of bread is the ingredients used. The type and quality of flour used can affect the texture, flavour, and nutritional content of the bread. Different types of flour have different levels of protein and gluten, which can affect the bread's texture and rise. For example, bread made with high-protein flour will have a chewier texture and a higher rise than bread made with lower-protein flour.

Other ingredients, such as yeast, salt, sugar and fats, can also affect the quality of bread. Yeast is responsible for the bread's rise, while salt enhances the flavour and helps to control the fermentation process. Sugar can be added to improve the flavour and colour of the bread, while fats such as butter or oil can make the bread softer and more tender.

The baking process is also critical to the quality of bread. The temperature and humidity in the oven can affect the texture and crust of the bread. If the oven temperature is too low, the bread may not rise properly or may have a tough crust. If the temperature is too high, the bread may burn or have a dry, tough interior.

The length of time the bread is baked can also affect its quality. If the bread is undercooked, it may be gummy or have a raw interior. If it is overcooked, it may be dry and crumbly.

Finally, the handling and storage of the bread after it is baked can also affect its quality. If the bread is not allowed to cool properly, it may become soggy. If it is stored in a humid environment, it may become stale or mouldy.

In conclusion, bread quality is determined by a variety of factors, including the ingredients used, the baking process, and the handling and storage of the bread. By paying close attention to these factors, bakers can produce high-quality bread that is delicious and nutritious, while consumers can choose bread that meets their preferences for taste and texture.

Text 2: Low authoritarianism text

When you think about what matters most to you, how important do you think it is that we should value diversity? Whether it is trying different types of food, enjoying better coffee, or just doing things a bit differently, do you think that it’s good to be open to new influences?

Think about those who have come here from overseas. Many people in Britain value the ways in which immigrants have added to our culture, and appreciate the foreign students who bring fresh, new ideas to revitalise our universities and colleges.

The Office for Budgetary Responsibility says that having more young, healthy, tax-paying immigrants would allow the government to reduce the national debt and save money on interest payments. According to figures quoted by the government, immigrants from the European Union make a particularly positive contribution in terms of the tax they pay in.

Currently, according to the NHS , over a quarter of our hospital doctors come from overseas and about one in five of our nurses, sharing best practice from around the world. Our creative industries are also open to international talent.

Take Sonia, who’s from Poland. She came here five years ago to sell clothes in London’s Camden Market. She enrolled at one of London’s top design colleges and is now producing fashion that pushes the creative boundaries, using new and unusual materials in original ways.

She loved the way that drag queens and Bollywood were included in the Queen’s Jubilee. For her, it is important that our institutions are finally giving diversity a fair hearing, and that race and religion are becoming ever less important. She feels that fashion can dissolve boundaries and transform people. At home, her favourite television programme is ‘Glow Up’, where make-up artists compete to create extraordinary designs.

Sonia would like to stay in the UK and has applied for British citizenship. Her dream is to design a version of the classic Dr Martens boot that defies convention, and then to sell it internationally, using London as her global base.

But she is worried about whether the UK is still open to new businesses, new people and new ideas. She feels there is a question mark over her future. Will she continue to be welcome in the UK, and if so, for how long? What do you think?

Text 3: Authoritarianism-compatible text

When you think about what matters most to you, do you think it’s appropriate for people who make a positive contribution to be treated fairly and get the thanks and recognition they deserve?

Think about those who have come here from overseas. There has been a change in how they are perceived in the UK. The latest polls show that most British people think it’s a good thing if skilled immigrants come and fill gaps in the labour market.

The Office for Budgetary Responsibility says that having more young, healthy, tax-paying immigrants would allow the government to reduce the national debt and save money on interest payments. According to figures quoted by the government, immigrants from the European Union make a particularly positive contribution in terms of the tax they pay in.

We’ve all seen the chaos that happens when essential jobs go unfilled. We need people to make fuel deliveries, work in care homes and staff our National Health Service. Currently, according to the NHS , over a quarter of our hospital doctors come from overseas and about one in five of our nurses.

Take Sonia, who is from Poland. She came to Britain five years ago to work as an NHS nurse. She took on extended shifts to care for her patients. She thinks chatting with them about their lives is one of the greatest perks of the job.

She loved the Queen’s Jubilee last year, and the way in which the nation recognised the extraordinary contribution made by the people staffing our hospitals, schools and essential services. For her, it’s important to notice when people go above and beyond the call of duty.

## She chose to come here because she speaks excellent English and because she wanted to live in a safe, fair-minded country where hard work is rewarded. She is also a fan of British wildlife documentaries like “The Blue Planet”.

## Sonia would like to stay in the UK and has applied for British citizenship. But she is very worried when she sees so many of her EU nursing colleagues leaving to go back to their home countries. She feels there is a question mark over her future. Will she continue to be welcome in the UK, and if so, for how long? What do you think?

**YouGov 2023 results**


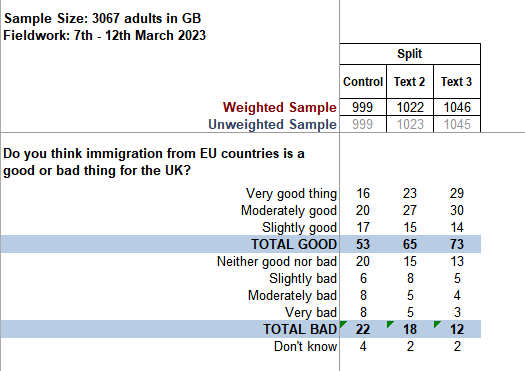
As referenced in the “Results” section, these YouGov results show the percentages of those exposed to each text who thought EU immigration was a good (“TOTAL GOOD”) thing for the EU. Text 3 is the authoritarianism-compatible text.

These results can be presented graphically as follows: **
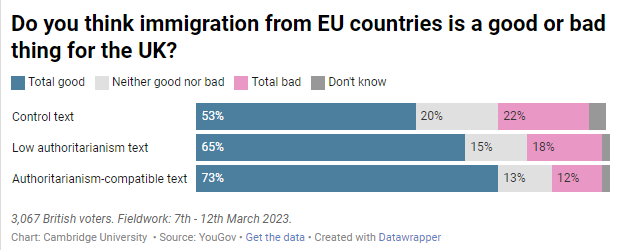
**

## **YouGov 2024 results**


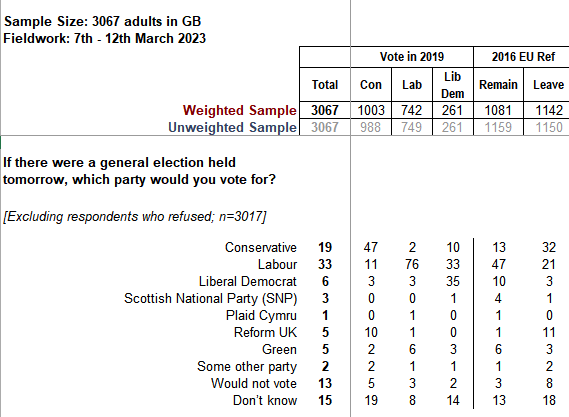
The following results show the political breakdown of those answering this survey.


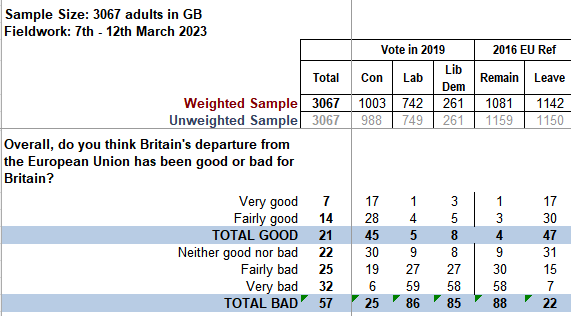


**Visualising responses based on authoritarianism scores**

The following graphs demonstrate that the response to the texts is non-linear.

**To what extent does the respondent share values with the immigrant? (High score = shares completely)**


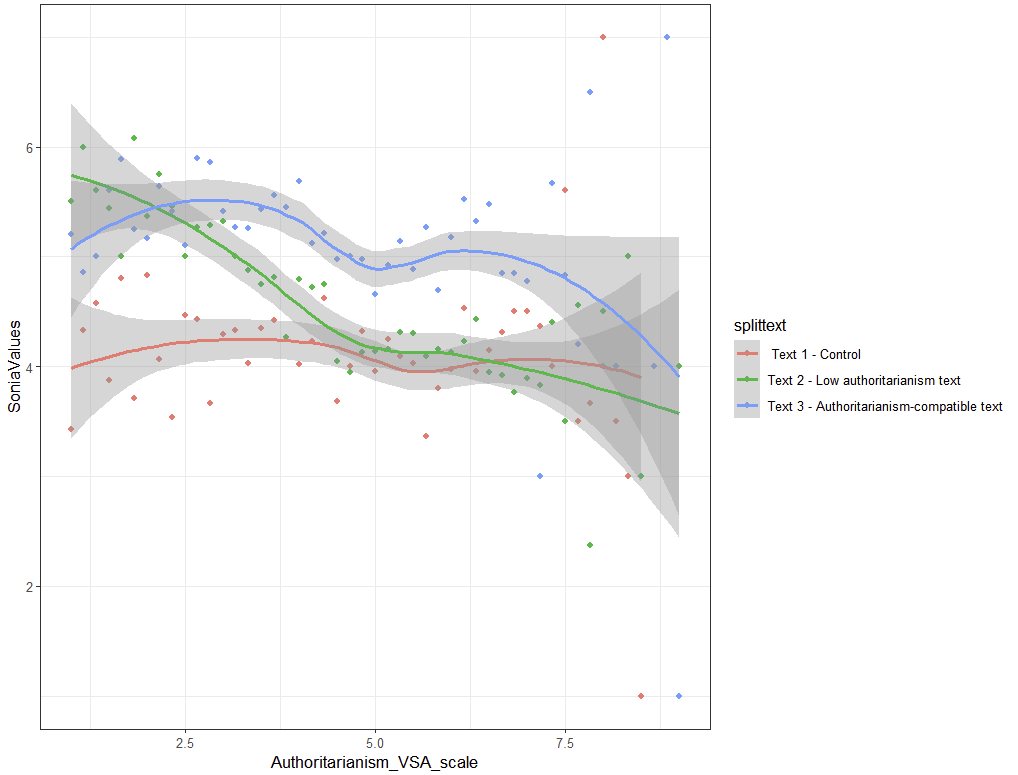


**Do you think EU immigration is a good or bad thing for the UK? (low score = good for UK)**


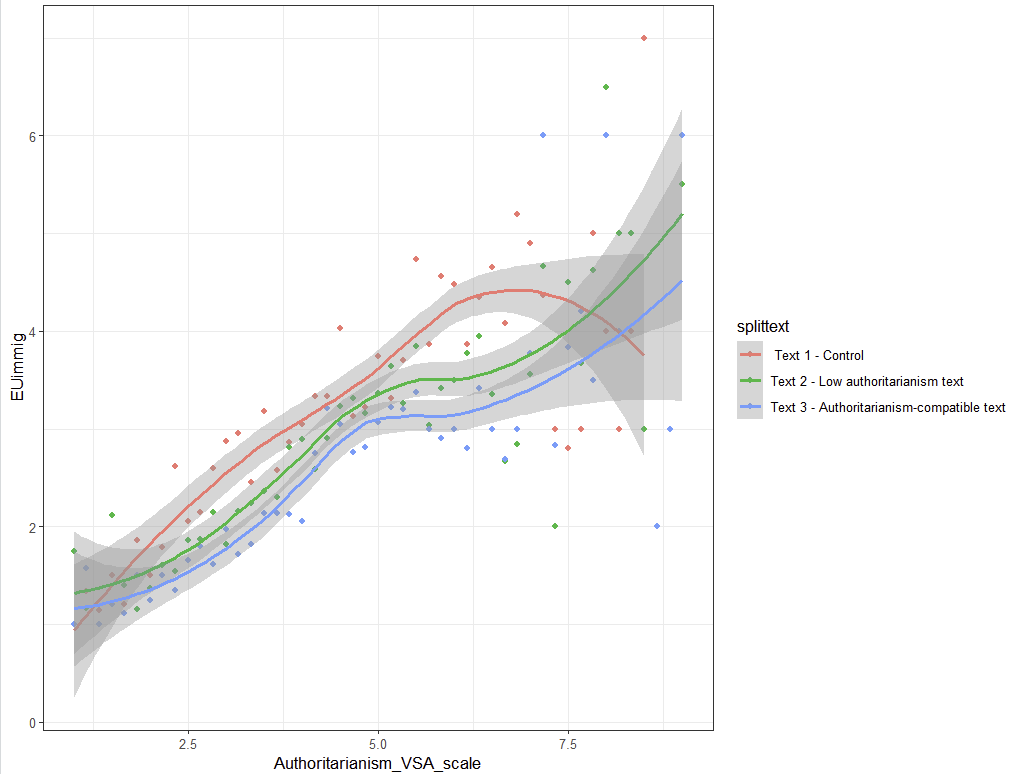


**Should many more or many fewer immigrants to allowed to come to the UK? (low score = many more)**


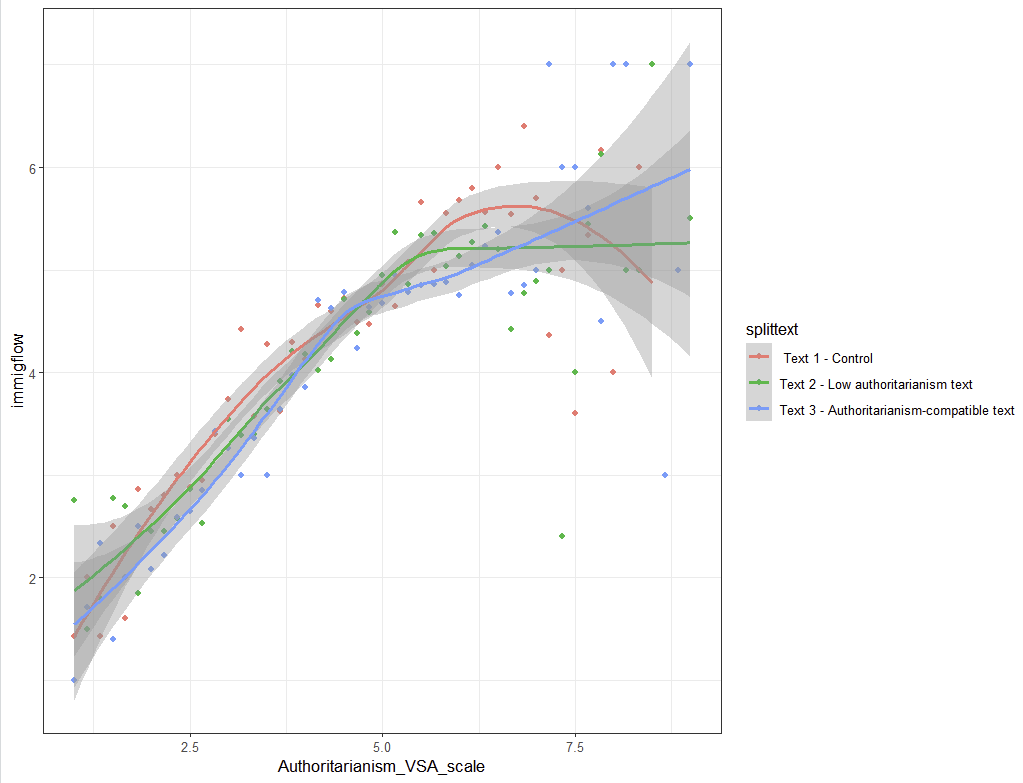


**Are there too many immigrants in the UK? (low score = too many)**


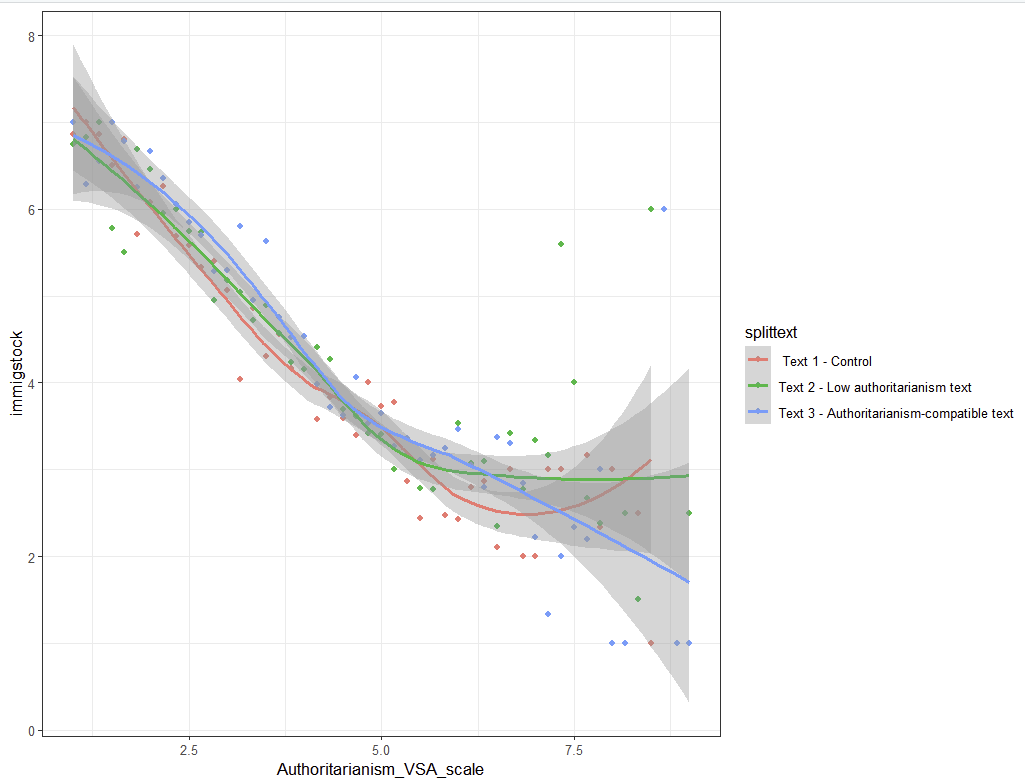

Supplement: Supplementary file 1 — Supplementary Information. [file 41598_2025_11491_MOESM1_ESM.docx]
